# Conformational Geometry Matters: The Case of the Low-Melting-Point Systems of Tetrabutylammonium Triflate with Fumaric or Maleic Acid

**DOI:** 10.3390/molecules29215093

**Published:** 2024-10-28

**Authors:** Simone Di Muzio, Fabio Ramondo, Oriele Palumbo, Francesco Trequattrini, Pascale Roy, Jean-Blaise Brubach, Annalisa Paolone

**Affiliations:** 1 Istituto dei Sistemi Complessi, Consiglio Nazionale delle Ricerche, UOS La Sapienza, Piazzale Aldo Moro 5, 00185 Rome, Italy; simone.dimuzio@ifn.cnr.it (S.D.M.); francesco.trequattrini@uniroma1.it (F.T.); annalisa.paolone@roma1.infn.it (A.P.); 2 Istituto di Fotonica e Nanotecnologie, Consiglio Nazionale delle Ricerche, Piazza Leonardo da Vinci, 32, 20133 Milano, Italy; 3 Department of Chemistry, Sapienza Università di Roma, Piazzale Aldo Moro 5, 00185 Rome, Italy; fabio.ramondo@uniroma1.it; 4 Department of Physics, Sapienza Università di Roma, Piazzale Aldo Moro 5, 00185 Rome, Italy; 5 Synchrotron SOLEIL, L’Orme des Merisiers, Départementale 128, 91190 Saint-Aubin, France; pascale.roy@synchrotron-soleil.fr (P.R.); jean-blaise.brubach@synchrotron-soleil.fr (J.-B.B.)

**Keywords:** low-melting-point systems, hydrogen bonding, density functional theory, infrared spectroscopy

## Abstract

For this article, the interaction of tetrabutylammonium trifluoromethanesulfonate (TBATFO) with either fumaric (FUM) or maleic (MAL) acid has been investigated. These acids are isomers and can be considered the trans and cis configurations of the same molecular geometry. When TBATFO is mixed with FUM, an eutectic point is obtained for a relative composition of 90-10 (molar ratio), with a melting point of ≈90 °C. If maleic acid is mixed with TBATFO, one obtains an inhomogeneous phase with the retention of a solid portion immersed in a liquid phase, even above 90 °C. DFT calculations helped to model the interaction between the components. It is suggested herein that TBATFO interacts more strongly with FUM than with MAL, due to possible interactions in two different sites for hydrogen bonding (HB) in FUM. In MAL, one of the HB sites is instead retained in the intramolecular interactions; therefore, fewer sites are available for intermolecular interactions. Infrared spectroscopy measurements have confirmed this scenario, in which the hydrogen bonds of the acid molecules are replaced by HB between the acid and the ionic couple: for both kinds of mixtures, the vibration region of the OH bonds is strongly affected by mixing. However, in the case of FUM, the vibrations of the SO_3_ group of the TFO anion are displaced, while they remain in practically the same frequency position in the case of MAL.

## 1. Introduction

The rising demand for solvents that offer both strong solvating capabilities and minimal environmental impact has steered research toward the development of alternative systems, such as deep eutectic solvents (DESs) [1]. These eutectic mixtures are typically formed by combining a hydrogen bond donor (HBD) with a hydrogen bond acceptor (HBA), resulting in a significant reduction in the melting point. DESs can be prepared easily by mixing non-toxic materials in specific molar ratios. Their ease of preparation allows the development of combinations with tunable physicochemical properties that can be tailored to specific applications [2,3,4,5]. By varying the HBD and/or HBA, in fact, it is possible to significantly alter the physicochemical properties of the obtained mixtures, such as hydrophobicity or hydrophilicity, density, viscosity, and melting point [6,7]. A comprehensive understanding of the formation mechanisms of these liquids and of the interactions governing their composing units is essential for accurately predicting and elucidating their different physicochemical properties. In particular, the term “deep” refers to a significant deviation from ideal behavior observed in these mixtures: unlike ideal eutectics, DESs exhibit pronounced non-ideal interactions between the HBD and HBA, which result in a substantial lowering of the melting point compared to the expected values, leading to the formation of a stable liquid phase. In other words, DESs exhibit melting points that are lower than those expected in ideal eutectic mixtures [8,9,10]. With the growing interest in eutectic systems, in recent years, several systems were proposed wherein the HBA is a tetrabutylammonium (TBA)-based salt, paired either with simple halides, such as bromide, chloride, or iodide [11,12,13,14] or with more complex anions, such as acetate [15]. These quaternary ammonium salts have been mixed with various HBDs, such as organic acids [15,16,17,18], alcohols [19,20], and imidazole-based molecules [21,22]. Many of the systems proposed thus far were based on TBA bromide or chloride: in these samples, it has been observed that the anions act as an HBA. For the present work, we investigated a more complex anion, specifically, trifluoromethanesulfonate (CF_3_SO_3_^−^, TFO). Interest in this ionic pair arises from the fact that TBATFO salt is widely used in various electrochemical applications: TBATFO has been proposed as an additive for Mg-ion batteries, with the aim of increasing the dissolution of magnesium salts and enhancing charge transport efficiency [23]. Furthermore, investigating the role of TFO as a hydrogen bond donor (HBD) is valuable due to its asymmetric structure, which features two potential hydrogen-bond-accepting sites: the SO_3_ and CF_3_ groups. For this research, TBATFO was mixed with two different dicarboxylic acids, namely, fumaric (FUM) and maleic acid (MAL). The organic acids used (Figure S1) can be considered structural isomers: trans and cis, respectively.

In our previous study [24], we showed that two choline-based ionic liquids, one with the monoanionic form of maleic acid and the other with the monoanionic form of fumaric acid, have significantly different melting points. Specifically, choline-H-maleate melts at 25 °C while choline-H-fumarate melts at 80 °C. The cis conformation of H-maleate allows for the formation of intramolecular H-bonds, which is not possible in the trans conformer.

The presence of intramolecular hydrogen bonds in choline-H-maleate, combined with the distinct structural arrangement of the components in the bulk phases of the two ionic liquids, significantly influenced their physicochemical properties, as indicated by the considerable difference in their melting points. The goal of the present study is to investigate two low-melting-point systems composed of TBATFO in combination with fumaric or maleic acid, with the aim of determining whether the distinct cis (fumaric) or trans (maleic) configurations of the HBD can influence the physicochemical properties of these systems. To achieve these aims, we determined the phase diagram of both systems using differential scanning calorimetry (DSC) measurements. Structural investigation was carried out by means of infrared spectroscopy measurements in the mid-IR and far-IR regions: the latter has been shown in several studies to be particularly useful for examining intermolecular interactions such as hydrogen bonding and dispersion forces [25]. Additionally, the systems were characterized, by means of density functional theory (DFT) calculations, to enhance the interpretation of the experimental results.

## 2. Results and Discussion

### 2.1. Determination of Eutectic Compositions by DSC Measurement

The range of compositions that correspond to the formation of the eutectic components was investigated by means of DSC measurements.

**Figure 1:**
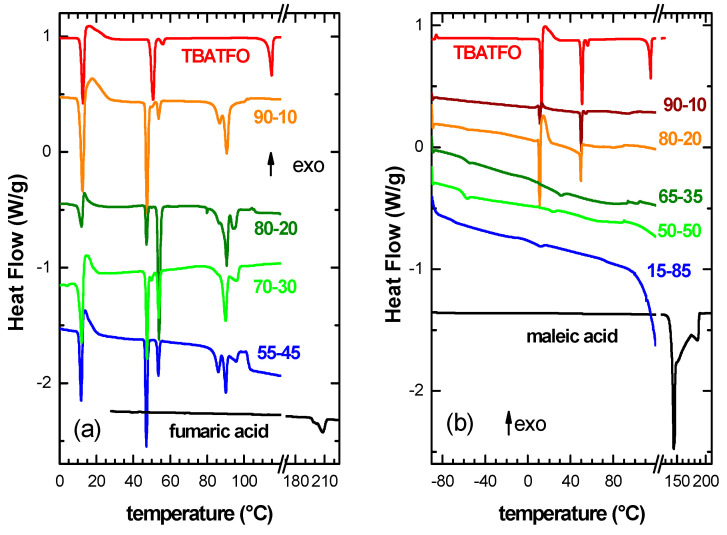
Differential scanning calorimetry measurements collected during heating for TBATFO, fumaric acid (panel (**a**)), maleic acid (panel (**b**)), and their mixtures.

Figure 1 reports the DSC measurements of the pure components and of the two series of mixtures between TBATFO and fumaric acid (panel a) or maleic acid (panel b). Pure TBATFO undergoes a melting process around 115 °C. A visual inspection indicated that this sample was certainly solid at around 80 °C. Therefore, the three additional endothermic processes that were present around 13, 50, and 56 °C should be ascribed to solid–solid phase transitions [19]. Fumaric (maleic) acid exhibited a structured endothermic peak between 190 and 215 °C (130 and 190 °C), which was due to the superposition of melting and the subsequent decomposition (as obtained from the TGA measurements in Figure S2).

All the mixtures with fumaric acid displayed endothermic peaks below 60 °C, which, in pure TBATFO, can be attributed to solid–solid transition, even though they are displaced by a few degrees to lower temperature values. None of them show the melting process of TBATFO, but rather a multistep melting process between 85 and 100 °C. The eutectic composition is supposed to display a single melting process, and the composition that most resembled this definition was that composed of a molar ratio of 90% TBATFO and 10% fumaric acid (Figure 1a).

Concerning the mixtures with maleic acid (Figure 1b), pure MAL exhibited a broad and complex endothermic peak between 150 and 200 °C, which included the melting process and partial decomposition (TGA measurements, Figure S2). The mixtures with compositions of 90-10 and 80-20 show DSC traces similar to that of pure TBATFO, even though one cannot see the melting process of the ionic salt, but instead show a broad endothermic peak at around 80 °C. The compositions between 65-35 and 15-85 display two broad endothermic processes, one around 40 °C and the other around 80 °C. It must be noted that these samples, when heated to around 90 °C, still present a solid component inside an apparent liquid phase. The lowest temperature at which the high-temperature features are present in the mixtures with maleic acid is around 70 °C for the 50-50 sample. In the following sections, we will indicate this sample as the eutectic of the system TBATFO–maleic acid for simplicity, even though it seems not to be a homogeneous phase. This is clearly different from the mixtures of TBATFO with fumaric acid, which yielded liquid phases when heated to around 90 °C.

In previous papers, the eutectic composition could be analytically determined according to previous knowledge of melting temperatures and the enthalpy of the pure components [19,26]. However, in the present case, the determination of the melting points and enthalpies of both acids is particularly difficult because the melting process is superimposed on the decomposition. Therefore, we only base our present study on a purely experimental approach.

### 2.2. Interaction Models by Quantum Chemistry

**Figure 2:**
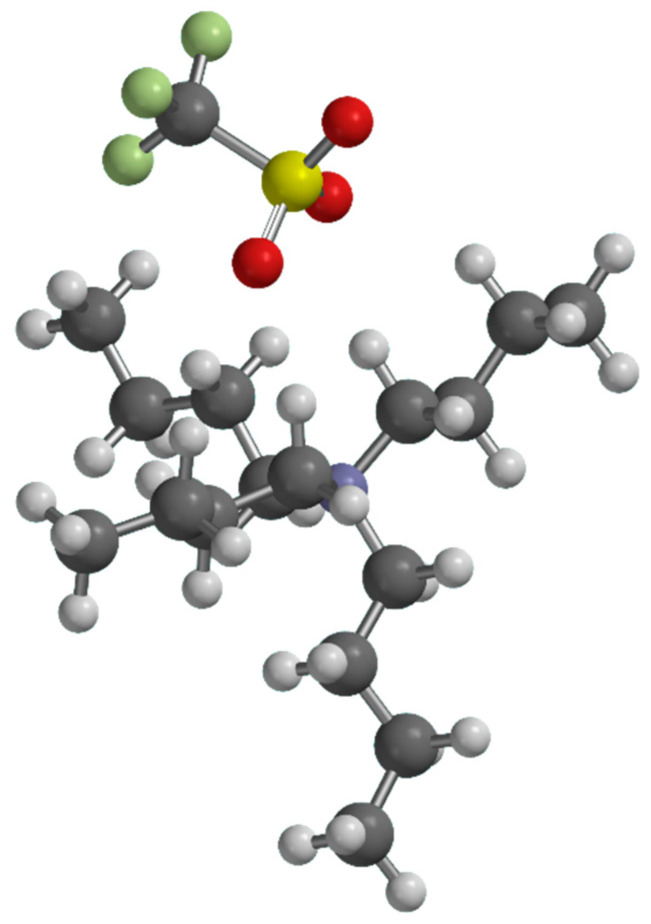
The TBATFO ion pair structure. White spheres are oxygen atoms, grey ones are carbon atoms, the yellow one is sulfur and the green ones are fluorine.

**Figure 3:**
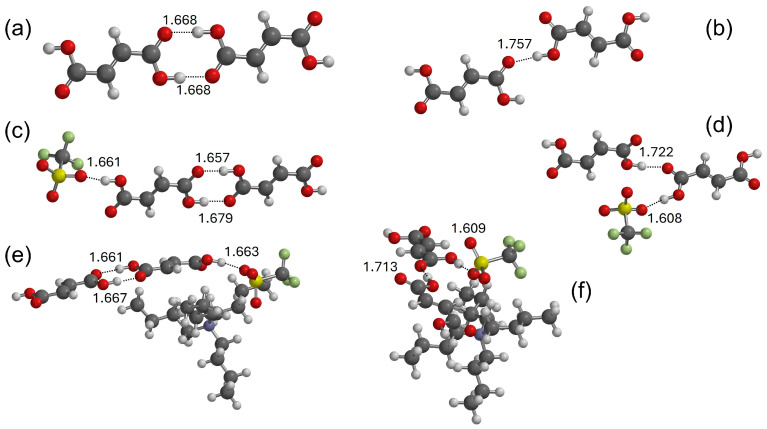
Structures of the closed and open dimers of FUM ((**a**) and (**b**), respectively), along with their complexes with TFO (**c**,**d**) and with TBATFO (**e**,**f**) (distances in Å). The color code is the same used in Figure 2.

**Figure 4:**
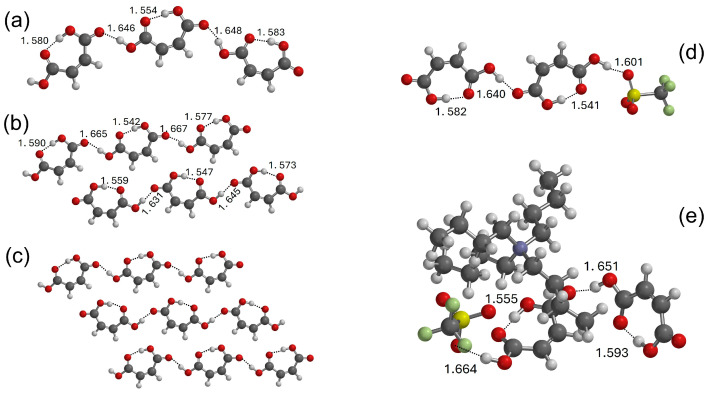
Structures of the oligomers of the MAL trimer (**a**), hexamer (**b**), and nonamer (**c**), and the TFO-MAL (**d**) and TBATFO-MAL (**e**) complexes (distances in Å). The color code is the same used in Figure 2.

The interactions between the ionic couple and the acids were investigated computationally by means of DFT calculations. Initially, we simulated the pure components, then we extended the calculations to some of the mixtures. The optimized structure of TBATFO is shown in Figure 2. Based on the previous literature about its crystal structure [27,28], the structural features of fumaric acid (FUM) were studied by considering four centrosymmetric oligomers: the dimer (Figure 3a), tetramer (Figure S3b), hexamer (Figure S3c), and octamer (Figure S3d), all with C_2h_ symmetry, and by considering an open dimeric structure (Figure 3b) where the monomers are connected by a single OH⋅⋅⋅O hydrogen bond. The self-aggregation of maleic acid (MAL) was considered for the trimer, the hexamer, and the nonamer depicted in Figure 4a–c, respectively: they are fragments of chains where the maleic acid molecules are connected by single hydrogen bonds and each chain is aligned to form planar layers, as reported after previous crystallographic measurements [29,30].

Some models have been proposed herein to evaluate the intermolecular interactions between different components in the mixtures. The interactions between TFO and acid have been described as a series of complexes wherein TFO is coupled with a single FUM (Figure S4a) or MAL (Figure S5a) molecule, with a dimer of FUM (Figure 3c,d) or MAL (Figure 4d) and with a trimer of FUM (Figure S4b or Figure S5b). The progressive increase in the number of acid molecules in such complexes allows us to investigate how the interaction of the negative head of the anion TFO with carboxylic acid is affected by the clustering of the acid component. The role of the cation in the aggregation process is then considered by adding a TBA cation to each TFO: the geometry of some TBA-TFO-acid complexes has been optimized and their structures are depicted in Figure 3e,f (TBA-TFO-FUM) and Figure 4e (TBA-TFO-MAL).

The crystal structure of FUM has been the subject of various studies [27,28]: the molecules are linked together by infinite chains of double hydrogen bonds between carboxylic groups, as found in several organic acids, and form planar layers. The structural features of a single layer can be obtained, starting from the centrosymmetric dimer reproduced in Figure 3a, where the equivalent hydrogen bonds show O⋅⋅⋅H distances of 1.668 Å. The double HB chain observed in the crystal has been simulated here by *C*_2h_ symmetry oligomers of progressively larger size: the tetramer, the hexamer, and the octamer, as shown in Figure S3. It is interesting to note that the H⋅⋅⋅O hydrogen bond distances of the central pair of molecules (1.666 Å for the tetramer, 1.666 Å for the hexamer, and 1.667 Å for the octamer) are very close to the value calculated for the dimer. This suggests that the centrosymmetric dimer, although very small, could provide a reasonable description of the structural features of the fumaric acid molecules in the crystal and of its vibrational spectrum. Therefore, notwithstanding the fact that we calculated the vibrational frequencies for all the oligomers proposed here, we employed the infrared spectrum calculated for the dimer to simulate the vibrational features of FUM before mixing. The picture could be more complicated upon the addition of a second component: the interaction with TFO anions could lead to the fragmentation of the double HB chains and to local structures where the acid molecules are still coupled, but by only one hydrogen bond. The second dimer reproduced in Figure 3b has, therefore, been proposed to consider this possible interaction pattern. The hydrogen bond in this open structure dimer is definitely weaker, as suggested by the longer O⋅⋅⋅H distance (1.757 Å). The AIM analysis of the two dimeric structures is summarized in Table 1: at the bond’s critical point (*r*_c_), localized on the O⋅⋅⋅H distance, the values of the electron density, ρ(*r*_c_) and its Laplacian, ∇^2^(*r*_c_), confirm that molecules form stronger HBs when they are paired through cyclic structures.

The addition of TBATFO leads to the establishment of interactions of FUM molecules with TFO anions and TBA cations that could give different coupling geometries. Among several interaction structures, we first focused on those involving only the TFO anion by considering the fact that the negative head of the triflate anion, SO_3_^−^, is a hydrogen bond acceptor that is stronger than the CF_3_ group [19]. The Fukui analysis carried out on TFO confirms that the nucleophilicity of oxygen atoms (+0.237 a.u.) is higher than that of fluorine atoms (+0.081 a.u.). Starting from the complex 1:1 (Figure S4a), we considered further models where TFO interacts with a FUM dimer (Figure 3c) and with a FUM trimer (Figure S4b), and we analyzed how the OH⋅⋅⋅OS hydrogen bond is affected by the model size. It is interesting to observe that the O⋅⋅⋅…OS distance is substantially unchanged in the three models (1.656 Å for the 1:1 complex, 1.661 Å for the 1:2 complex, and 1.660 Å for the 1:3 complex). For the 1:2 complex, we considered two different interaction couplings: the structure reproduced in Figure 3c takes into account a situation in which TFO interacts with FUM molecules that are still part of a double HB chain, whereas the structure in Figure 3d is a complex between the open dimer of FUM and TFO, and describes a local arrangement where anions interact with acid molecules coming from the breaking of the double HB bond chain. The hydrogen bond distances shown in Figure 3 and the AIM analysis reported in Table 1 suggest that the interaction with the TFO anion does not drastically change the geometry and the electronic features of the double HB coupling (Figure 3c), whereas it does produce a small strengthening of the single hydrogen bond (Figure 3d). In addition, the interaction with TFO seems to be stronger when the acid molecules break the double hydrogen bond chain, as suggested by the OH⋅⋅⋅OS distance (1.661 Å for the structure in Figure 3c and 1.608 Å for the structure in Figure 3d).

The AIM analysis results are consistent with the natural bond orbital (NBO) analysis, which is summarized in Table S1. This was carried out by considering all possible interactions between filled donor and empty acceptor NBOs and estimating their energy importance using second-order perturbation theory. The charge transfer between the lone pair (LP) of oxygen (proton acceptor) and the antibonds of the proton donor (σ*OH) correlates with the strength of the OH⋅⋅⋅O interaction and can provide a description of the contribution of hydrogen bonding to the total energy of the complex. Our NBO analysis proves that the oxygen lone pair donation to the anti-bonding orbital of the OH bond (LP(O) → σ*OH) gives strong stabilization (24.6 kcal mol^−1^) to the centrosymmetric FUM dimer; its interaction with TFO produces appreciable asymmetry on two hydrogen bonds, whereas the LP(O) donation of TFO toward the antibonding of the OH bond of FUM gives lower stabilization (12.6 kcal mol^−1^). The opening of the ring structure of the FUM dimer lowers the stabilization energy of only one LP(O) → σ*OH donation of the complex (10.5 kcal mol^−1^), whereas the interaction with TFO strengthens the FUM pairing, as predicted by AIM analysis.

Finally, the role of the cation was evaluated by adding a TBA cation in both the complexes with TFO; the optimized structures are reproduced in Figure 3e,f. The examination of their geometries indicates that the positive nitrogen of TBA is oriented toward the negative head of the triflate anion, SO_3_^−^, suggesting that coupling between cations and anions is driven, undoubtedly, by electrostatic forces, although this orientation suggests the possibility of aligning some CH groups of TBA to make CH⋅⋅⋅O and CH⋅⋅⋅F interactions (the shortest CH⋅⋅⋅O and CH⋅⋅⋅F distances are 2.275 Å and 2.578 Å, respectively, for the structure in Figure 3e and 2.233 Å and 2.709 Å, respectively, for the structure in Figure 3f). The main result is that the presence of the TBA cation does not significantly change the strength and geometry of the interaction of acid molecules with themselves and with TFO anions.

If the *trans* configuration of the C=C bond of fumaric acid allows it to form infinite chains of double HBs, the *cis* isomer, i.e., maleic acid, forms infinite chains of single HBs, as one of the protons is involved in an intramolecular hydrogen bond [29,30]. Each molecule shows an intramolecular hydrogen bond (an O⋯O distance equal to 2.50 Å [30]), due to the closing of a six-membered ring, and is linked to the neighboring molecules by longer intermolecular hydrogen bonds (an O⋯O distance equal to 2.64 Å [30]) to form linear chains; each row is interlinked by weaker hydrogen bonds (an O⋯O distance equal to 2.98 Å [29]) to form layers. A simulation of MAL molecules in the crystal environment is suggested by the complexes shown in Figure 4. The trimer of Figure 4a can be considered a fragment of a linear chain in which the molecules are connected by a single intermolecular HB, whereas the hexamer of Figure 4b and the nonamer of Figure 4c are models that show how the molecules can align their chains to form planar layers. The hexamer demonstrates how two linear chains are aligned in the crystal, and the nonamer shows the orientation of three linear chains. The geometries of all the oligomers have been optimized by assuming planar structures. An analysis of the O⋅⋅⋅O distances calculated for the largest cluster, the nonamer, confirms the presence of an intermolecular hydrogen bond along the linear chains (average value of 2.581 Å) and the O⋅⋅⋅O and CH⋅⋅⋅O contacts between chains (2.986 Å and 2.338 Å), along with the intramolecular hydrogen bond for each molecule (an O⋅⋅⋅O distance equal to 2.581 Å). All the values are in substantial agreement with the experimental values [29,30]. The values of the OH⋅⋅⋅O hydrogen bond distances calculated for the oligomers herein proposed are shown in Figure 3a,b for the trimer and hexamer, respectively, and in Figure S5 for the nonamer. The nature of the interactions between MAL molecules in the planar layers has been evaluated using the NCI approach, and the RDG isosurface is reproduced in Figure S6. The blue spikes localized in the OH⋅⋅⋅O region correspond to hydrogen bonding interactions and confirm the presence of intramolecular HBs and intermolecular HBs, which link the molecules in linear chains. The green surface localized in the region between the chains suggests that the association of chains to form layers is largely driven by CH⋅⋅⋅O interactions. The scatter plot using promolecular density, reproduced in Figure S7a, shows spikes with large negative values (−0.06 a.u.) that indicate the presence of strong hydrogen bonding in the MAL nonamer, along with appreciable van der Waals contributions. A comparison with the RDG scatter plot of the FUM octamer depicted in Figure S7b shows a negative spike at values higher than −0.06 a.u. because FUM does not show the strong intramolecular hydrogen bond of MAL, whereas the green spike is barely pronounced.

As observed with the FUM-based mixtures, the addition of TBATFO to maleic acid can lead to the breaking of the intermolecular HB network and to the formation of SO⋅⋅⋅HO interactions with the SO_3_^−^ groups. Following the procedure adopted for FUM, we investigated three complexes in which TFO interacts with one MAL molecule (Figure S8a), a MAL dimer (Figure 4d), and a MAL trimer, as shown in Figure S8b. Since the structural features of the complexes are not drastically dependent on the size of the model, we discuss the interaction of TFO with MAL by adopting the complex depicted in Figure 4d, where a maleic acid molecule is paired by an HB with TFO and a second acid molecule. The strength of the intermolecular interactions was once again analyzed using AIM calculations, and the results are reported in Table 2. The electron density features and intermolecular distances indicate that the intramolecular hydrogen bond is stronger than the intermolecular hydrogen bond, both before and after the interaction with TFO. This result is in agreement with the NBO analysis reported in Table S2, where we can observe the largest LP(O) → σ*OH donation from the OH bonds involved in intramolecular hydrogen bonds. Consistently, the RDG scatter plot of the complex of TFO with the MAL dimer (Figure S7c) shows that there are still spikes at low values that are close to those observed for the MAL nonamer, which are caused by the presence of strong intramolecular hydrogen bonding. In addition, the AIM analysis could suggest that MAL forms a hydrogen bond with the TFO anions that is slightly stronger than those formed with the acid molecules; however, the presence of the TBA cation, simulated by the complex depicted in Figure 4e, gives O⋅⋅⋅O very similar intermolecular distances as TFO-MAL and MAL-MAL. Apart from this small effect, the presence of the TBA cation does not profoundly change the structural features of the complex of TFO with MAL that is considered in Figure 4d, as already observed in the complexes with FUM. Based on these considerations, in the following section, we will discuss the infrared spectra of the mixtures by analyzing the theoretical spectra calculated for those complexes with TFO without including the TBA cation.

### 2.3. Interaction Between the Components by Computed and Experimental Infrared Spectra

In this section, the spectra measured for the starting materials and the mixtures will be compared and the changes observed upon mixing will be discussed, with the aim of investigating how the different components interact in the mixtures. The main absorptions have been assigned on the basis of the spectra calculated for the molecular models at the ωB97X-D/6-311++G** level, which are described in the previous section.

**Figure 5:**
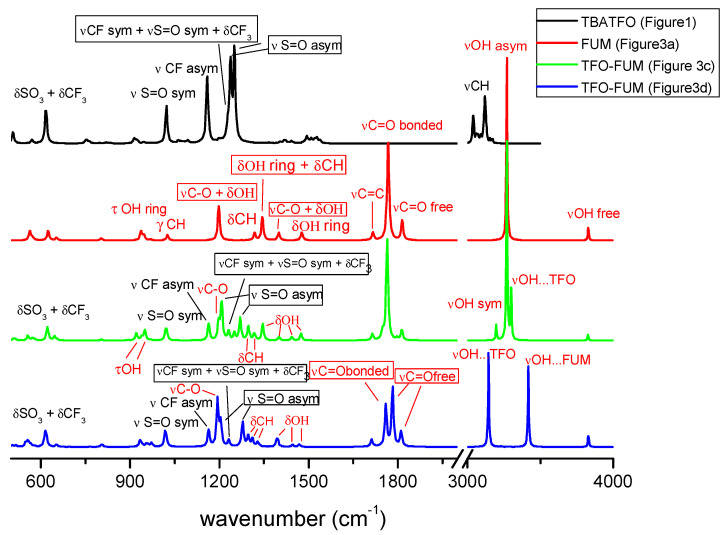
Infrared spectra calculated for the TBATFO ion pair (Figure 1), the FUM dimer (Figure 3a), and the TFO-FUM complexes shown in Figure 3c,d.

Starting the discussion with the TBATFO-FUM mixtures, we compare the infrared spectra in Figure 5 that were calculated for the models to simulate the pure components and mixtures. For TBATFO, we assume the ion pair to be a reasonable model for the pure compound, whereas, for FUM, we consider the smallest oligomer between the various clusters proposed (Figure 3a) to be a valid model for simulating the spectrum of pure FUM. The infrared spectra of oligomers are compared in Figure S9, and they show that the absorption bands do not change appreciably along with the size of the oligomer. The spectrum of the mixtures has been simulated by the TFO-FUM complexes, in which a TFO anion interacts with a closed FUM dimer (Figure 3c) or with an open FUM dimer (Figure 3d). The presence of TBA cations has been taken into account for each TFO-FUM complex and the infrared spectra have been calculated for the TBA-TFO-FUM complexes depicted in Figure S10 in the Supplementary Materials. Since the spectral changes are localized mainly on the bands of FUM and TFO components and are only marginally on those of TBA, in Figure 5, we depict the spectra of the TFO-FUM complexes without the TBA cation and we show the assignment of the main bands.

In the high-frequency region, we expected the OH stretching vibrations of FUM to be significantly perturbed upon mixing. The centrosymmetric dimer shows a single absorption, i.e., the asymmetric stretching mode of the OH groups involved in the HB cyclic structure; the interaction with TFO causes a lowering of the local symmetry of the dimer, and new bands are observed. In particular, when TFO interacts with a double-hydrogen-bonded fumaric dimer, as occurs in the complex depicted in Figure 3c, we would expect to observe the OH symmetric stretching mode, although at a low intensity, and the stretching of the OH group bonded to TFO. However, the spectrum depicted in Figure 5 indicates that its frequency is not too far from that of the OH asymmetric stretching, which is in agreement with the geometry of the complex where the FUM-FUM and FUM-TFO hydrogen bonds show very close distances. In contrast, when the double HB chain of FUM is broken and FUM can form a single HB with TFO and FUM, as in the complex shown in Figure 3d, the OH stretching vibrations are well separated and we can predict two absorptions: at a lower frequency, we expect the stretching of the OH group bonded to TFO, while at a higher frequency, we expect that of the OH group bonded to the second FUM molecule.

**Figure 6:**
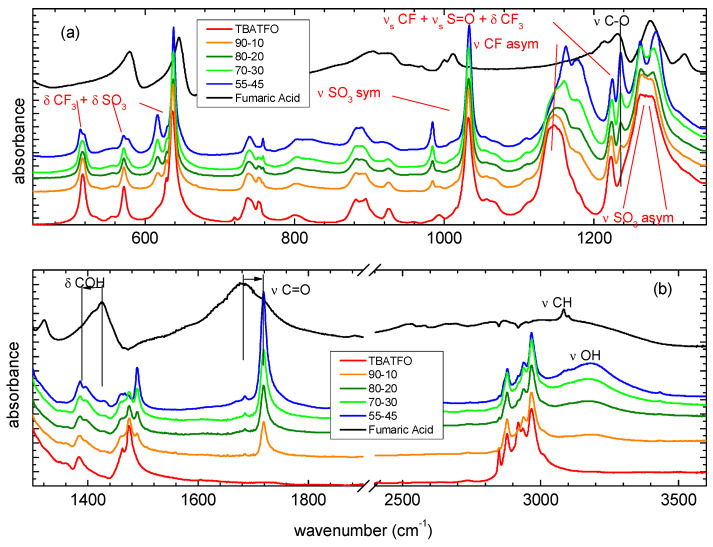
Infrared spectra of TBATFO, fumaric acid, and their mixtures in various compositions in the frequency ranges of 420–1300 cm^−1^ (panel (**a**)) and 1300–3600 cm^−1^ (panel (**b**)).

Figure 6 shows the experimental infrared spectra that were measured for pure components and mixtures at different molar ratios. The line shape of the high-frequency region is very complex and shows a series of absorptions below 3000 cm^−1^, a large band ranging from ≈3000 cm^−1^ to ≈3400 cm^−1^, and a fairly sharp peak at 3080 cm^−1^ (Figure 6b). The congestion of the bands in this region reflects the complicated underlying interactions and phenomena involving the OH group [31]. In addition, inhomogeneous broadening can be the result of the presence of different dimeric structures [32]. Although the CH stretching absorptions are predicted to be very weak according to our DFT calculations, in agreement with the spectra measured for FUM isolated in a solid nitrogen matrix [33], the sharp absorption at 3080 cm^−1^ that was measured in the IR spectrum of solid FUM is present, with a very similar line shape that is also in the Raman spectrum [34]; therefore, it can be assigned to the CH stretching mode. The stretching modes of the OH groups of FUM molecules were hydrogen-bonded in a crystal state and are responsible for the remaining and complex series of broad absorptions. The isomerism by proton transfer expected for FUM crystals could explain the presence of OH stretching absorptions below 3000 cm^−1^ [31]. The spectral pattern is deeply changed by adding TBATFO: the complex series of absorptions below 3000 cm^−1^ disappears, and all the mixtures show only quite large absorption at about 3180 cm^−1^, with an intensity that progressively increases with FUM concentration. This blue shift of OH stretching is consistent with the theoretical spectra of the TFO-FUM complex, where the double HB of the acid molecules is broken and the OH vibration moves at a higher frequency. The CH stretching modes of TBA give a series of strong and sharp absorption values below 3000 cm^−1^; however, it is very hard to appreciate changes in this region by comparing the spectra of pure TBATFO and its mixtures.

More interestingly, we observe marked differences in the medium-frequency region, as depicted in Figure 6. The C=O stretching mode of the carboxylic group gives a very strong and large absorption in the spectrum of solid FUM, with maximum absorption at about 1680 cm^−1^ and a very weak shoulder at 1720 cm^−1^ (Figure 6b). The line shape changes markedly after adding the second component: by comparing the mixtures at different TBATFO:FUM molar ratios, we observe a sharp absorption at 1718 cm^−1^ and a second small peak at 1685 cm^−1^. Such a weak absorption is found in the Raman spectra as a strong peak at 1687 cm^−1^ [34]; therefore, it can be assigned to the C=C stretching vibration. The changes observed for the C=O stretching bands suggest that the FUM molecules in the mixtures could be involved in interactions with TFO by OH⋅⋅⋅O=S bonds and simultaneously with FUM by a double HB, as in the complex shown in Figure 3c, or by a single HB, as in the complex shown in Figure 3d. The DFT results indicate that the opening of the ring HB dimeric structure of FUM gives a blue shift to the C=O stretching absorptions, which is in agreement with the spectra measured for the mixtures. Other vibrational modes of the carboxylic group that can be perturbed by the HB are the OH bending (δCOH), the CO stretching (νCO), and the CO torsion (τCO) modes. In the region of the δCOH vibrations, the spectrum of solid FUM shows a strong absorption at 1425 cm^−1^, with a shoulder at 1407 cm^−1^ and a weaker band at 1320 cm^−1^ (Figure 6). It is interesting to observe that the absorption at 1320 cm^−1^ disappears, whereas that at 1425 cm^−1^ drastically weakens when the second component is added to FUM and a new band appears at about 1400 cm^−1^. Our interaction models predict a series of vibrational modes involving the OH and CH bending contributions in this region, which are appreciably coupled with each other and give spectral patterns that are different for the various interaction models. The spectral changes observed for these bands are highly indicative of the fact that the interaction of FUM with TFO undoubtedly involves the OH group, forming hydrogen bonding. In addition, the redshifts observed for these absorptions, along with the blueshifts measured for the OH and C=O stretching bands, are consistent with the fact that the dimerization of acid is depressed by adding TBATFO.

In Figure 6a, we compare the spectra in the medium-frequency region where the absorptions of the triflate ion are expected. The stretching modes of the SO_3_^−^ and CF_3_ groups give indications as to the nature and strength of the interactions with FUM: the antisymmetric SO stretching (νS=O asym), double-degenerated in the isolated triflate anion, is split into two bands when SO_3_^−^ interacts with TBA or with FUM since the *C*_3v_ local symmetry of the SO_3_ group is lowered; the width of the splitting may be related to the strength of the interaction. As predicted by the DFT calculations, the TBA cation has a low-density charge, and coupling with TFO involves the whole SO_3_^−^ group and causes weak asymmetry. The band that is measured for pure TBATFO is, therefore, quite large, with splitting estimated to be about 10 cm^−1^. In contrast, when TBA is coupled with high-density charge cations, such as lithium, the splitting is definitely larger (about 100 cm^−1^) [35]. The band continues to be split (1264 and 1283 cm^−1^) in the presence of FUM (Figure 6a); the width of the splitting is increased compared to that of pure TBATFO, but it is lower than those predicted from our interaction models, which are significantly more marked (65 cm^−1^ for the complex of Figure 3c, and 74 cm^−1^ for the complex of Figure 3d). This can be explained by considering the fact that the interaction models are static and the intermolecular interaction is described by an extreme localization of the OH⋅⋅⋅OS hydrogen bond, without any dynamic effect. This suggests that there are interactions between TFO and FUM, but their strength is overestimated by our models. It is interesting to observe that, as for the SO_3_^−^ group, the asymmetric stretching of the CF_3_ group (νCF asym) also shows quite a large absorption at 1147 cm^−1^, suggesting a tendency to split the mode into two components; the splitting is clearly better resolved in those mixtures where two spectral components are measured at 1161 and 1177 cm^−1^. In addition, we note that this band moves from 1147 cm^−1^ in TBATFO to a higher frequency upon mixing, suggesting that the interactions of TFO with FUM could involve the group of SO_3_ through the OH⋅⋅⋅O(S) hydrogen bond, as well as the group of CF_3_ through CF⋅⋅⋅H interactions. The symmetric S=O stretching (νS=O sym) yields strong and sharp absorption at 1033 cm^−1^ in TBATFO and its mixtures at all FUM concentrations; similarly, the sharp peak at 1225 cm^−1^, assigned to a complex vibration involving the symmetric stretching of CF_3_ and SO_3_ and the bending of CF_3_, is found to be practically unchanged before and after mixing. The bending modes of SO_3_ and CF_3_ are strongly coupled and give sharp absorptions at 637, 572, and 514 cm^−1^, being substantially unchanged by mixing.

In addition, the spectra of the mixtures show an additional and sharp absorption at 1238 cm^−1^, also observed before mixing in the FUM solid spectrum. The DFT calculations suggest assignment to some vibration involving νC-O, δCCH, and δCOH contributions. A similar assignment may be proposed for the strong and large absorption observed in the solid FUM spectrum at 1275 cm^−1^, although it is hard to appreciate the presence of this band in the mixtures since their spectra show strong νS=O asym absorption at the same frequency.

**Figure 7:**
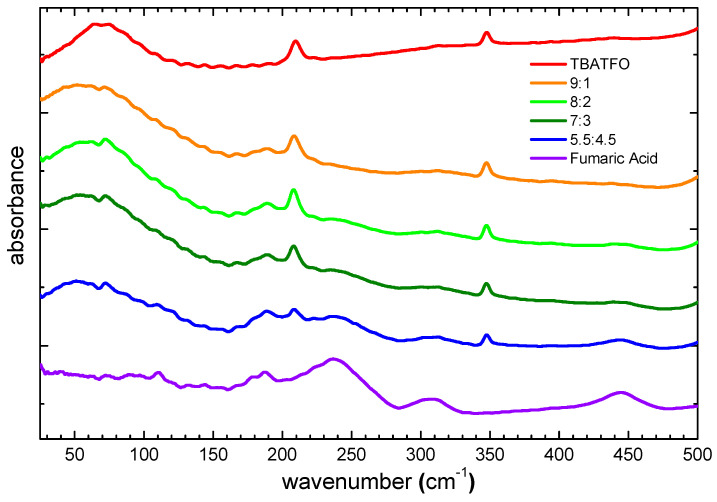
Far-infrared spectra at room temperature of TBATFO, fumaric acid, and their mixtures in various compositions.

Another interesting part of the infrared spectra is seen below 200 cm^−1^, where the vibrational modes of the hydrogen bonding and the dispersion forces are present [25,36]. Indeed, the occurrence of such absorption peaks has largely been investigated in ionic liquids, especially the protic ones, where the occurrence of HB is witnessed by the presence of absorption peaks around 50 cm^−1^ for the bending mode of HB and at around 120–150 cm^−1^ for the stretching vibration. In between these two peaks, additional absorption is usually found, centered around 70–80 cm^−1^, due to the dispersion forces among the tails of the ion chains [25,36,37]. In the case of the mixtures with fumaric acid (see Figure 7), no clear evidence of the bending and stretching bonds of HB is visible. This fact is not intuitive, but it can be explained in the pure acid as being due to the static nature of the interaction. Possibly, in the mixtures, the directionality of the HBs is not sufficient to give strong hydrogen stretching modes.

In pure TBATFO a well-developed peak around 70 cm^−1^ is observable. Due to the position of its maximum, this can be assigned to the dispersion forces among the alkyl chains of the cation. This peak shifts to a lower frequency (about 60 cm^−1^) in all mixtures, suggesting a reduction in the interactions among the cations. This can be explained by a possible reduction in the interactions between cations as they become more spatially separated due to the addition of the acid.

### 2.4. TBATFO-MAL Mixtures

**Figure 8:**
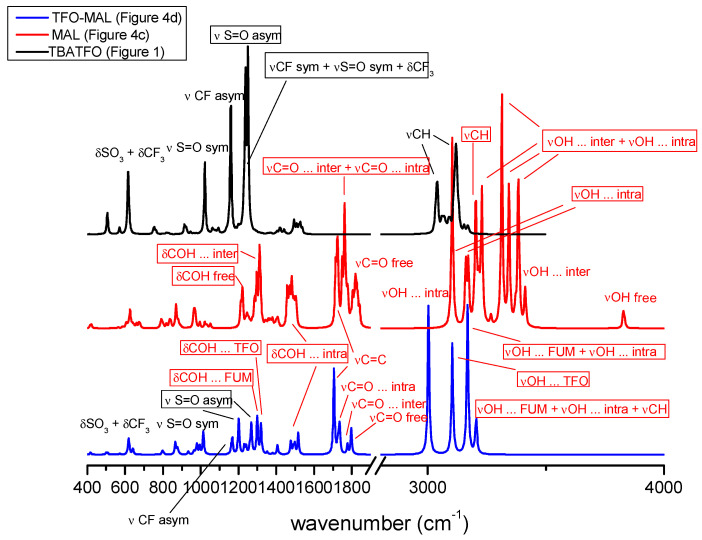
Infrared spectra calculated for the TBATFO ion pair (Figure 1), the MAL nonamer (Figure 4c), and the TFO-MAL complex (Figure 4d).

Figure 8 compares the infrared spectra calculated for the TBATFO ion pair (Figure 1), the MAL nonamer (Figure 4c), and the complex between TFO and two MAL molecules (Figure 4, panels d, and shows the assignment of the main bands. Regarding the mixtures with FUM, we report the spectra of TFO-MAL complexes without the TBA cation: the spectra of the TFO_MAL and TBA-TFO-MAL are compared in Figure S11. In the oligomer that is used to simulate pure maleic acid, the MAL nonamer, the high-frequency region shows an intense CH stretching band and a series of absorptions, due to the vibrations of the OH groups involved in intra- and intermolecular hydrogen bonds. The vibrations at a lower frequency are mainly localized on the OH groups that are intramolecularly hydrogen-bonded, whereas the remaining absorptions are due to the collective stretching vibrations of OH groups that are intramolecularly and intermolecularly hydrogen-bonded.

**Figure 9:**
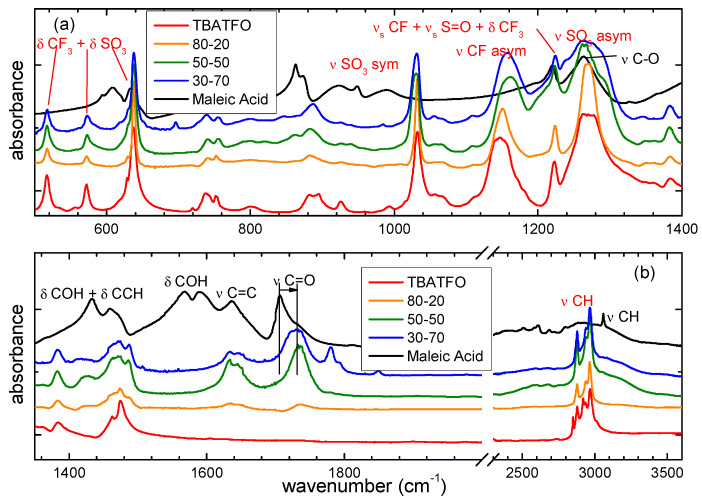
Infrared spectra of TBATFO, maleic acid, and their mixtures at various compositions in the frequency ranges of 500–1400 cm^−1^ (panel (**a**)) and 1350–3600 cm^−1^ (panel (**b**)).

Figure 9 shows the spectra measured for the pure components and their mixtures. The sharp peak at 3058 cm^−1^ can be assigned to the CH stretching mode, in agreement with the same band that was observed for solid FUM at 3080 cm^−1^. As for FUM, the OH stretching region of solid MAL also has a complex line shape, with broad absorption between 3100 and 2800 cm^−1^ and a series of bands below 3000 cm^−1^. The line shape of the OH stretching absorption of the crystalline MAL has been investigated in a previous study using molecular dynamics, considering the proton transfer by intramolecular as well as intermolecular HBs, and absorptions were predicted up to about 2300 cm^−1^ [38]. As for the FUM-based mixtures, the OH stretching absorptions of MAL change upon mixing, although less visibly. While the addition of TBATFO to FUM produces the appearance of a very distinct peak at a higher frequency and the disappearance of absorptions below 3000 cm^−1^, the mixing of TBATFO and MAL gives only a small shoulder at a higher frequency of the main peak of OH adsorption, which is indicative of the breaking of the HB network present in the crystalline MAL, but this does not markedly change the spectral region below 3000 cm^−1^, where weak absorptions continue to be observed. This is indicative of the fact that if, in the FUM-based mixtures, the presence of TFO contributes to breaking the strong double HB that pairs the molecules in the FUM crystal to form weaker FUM⋅⋅⋅TFO interactions, the MAL⋅⋅⋅TFO interactions formed in the base MAL mixtures may instead have a strength quite similar to that of MAL⋅⋅⋅MAL HB, as suggested by the structures discussed in the previous sections; therefore, they produce less marked spectral changes. In addition, the MAL nonamer structure reveals that the intermolecular HB network produces strong coupling between the vibrations of all the OH groups; such a coupling weakens when the HB chains are broken to form interactions with TFO, and the vibrations of OH groups, both intramolecularly and intermolecularly HB-bonded, are better separated. This means that in the mixtures, the acid molecules continue to have intramolecular HBs and to show absorptions below 3000 cm^−1^.

In the medium-frequency region (Figure 9b), we observe some bands of MAL that are substantially unchanged upon mixing, as with the absorption at 1634 cm^−1^, assigned to the C=C stretching mode, and other bands that move their absorption frequency when TBATFO is added. Two bands are expected for the C=O stretching modes: one is related to the C=O group, intermolecularly HB-bonded, measured at 1706 cm^−1^ in the solid MAL and blue-shifted to 1734 cm^−1^ by adding the second component, as already observed for FUM-based mixtures; the second one, related to the C=O group that is intramolecularly HB-bonded, measured at 1722 cm^−1^ for MAL isolated in a nitrogen matrix [33], could tentatively be assigned to the shoulder at 1734 cm^−1^ that we find again at the same frequency in the mixtures.

At a lower frequency, our interaction models predict a series of vibrational modes involving CCH bending (δCCH), COH bending (δCOH), and CO stretching (νCO): those vibrations with a large contribution of δCOH are more affected by changes in the molecular environment. For example, the bands measured at 1590 and 1586 cm^−1^ in the spectrum of solid MAL can be assigned to vibrations that are mainly localized on the OH group bonded by intramolecular HBs. It is interesting to observe that the spectra of the mixtures do not show any absorption in this region, in agreement with the fact that the strength of intramolecular hydrogen bonding may change from the pure component to its mixtures. Our interaction models suggest that intramolecular HBs are stronger for the complexes MAL-TFO and MAL-TBATFO than for any MAL oligomer; the δCOH vibrations should, therefore, increase their frequencies upon mixing, and the appearance of a new absorption at 1645 cm^−1^ in the mixtures could be interpreted in these terms.

Looking at the absorptions of triflate anion in the mixtures (Figure 9), we find the same spectral pattern being observed for the FUM-based mixtures. The symmetric stretching of the SO_3_^−^ groups (1030 cm^−1^), the vibrational mode with a mixed contribution of symmetric νCF_3_, νSO_3_, and δCF_3_ (1223 cm^−1^), and the bending modes of CF_3_ and SO_3_ (638, 573, and 517 cm^−1^) are found to be unchanged upon mixing. The antisymmetric νCF_3_ and νSO_3_ give quite broad absorptions in the MAL spectrum; while these bands are split in the mixtures with FUM, the splitting is not resolved in the mixtures with MAL. These spectral features indicate that the coordination of MAL to TFO causes an asymmetry of the SO_3_^−^ and CF_3_ groups that is less marked than that seen due to the coordination of FUM, suggesting that the MAL and TFO components have a spatial distribution driven by weakly directional interactions. Lastly, the spectra of the MAL-based mixtures show additional absorptions at 1293 and 1204 cm^−1^; they appear as shoulders of the main absorptions and are associated with vibrations involving C-O stretching. In the same spectral region, the spectrum of solid MAL shows two absorptions at 1223 and 1264 cm^−1^.

**Figure 10:**
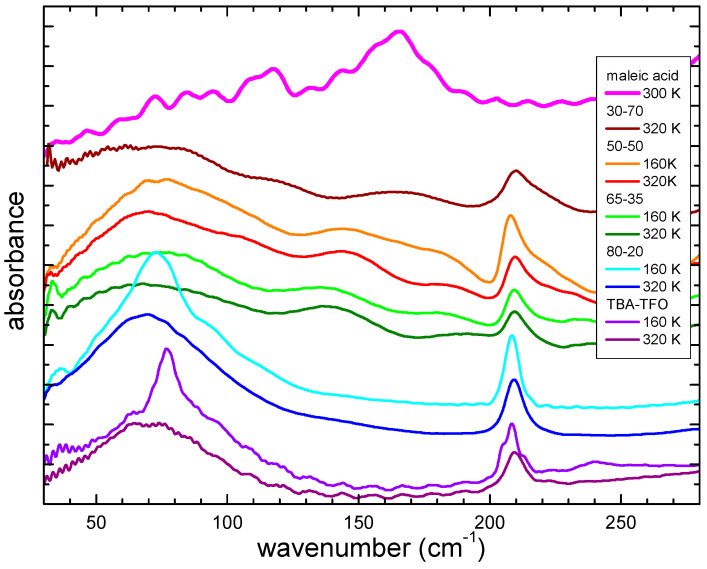
Far-infrared spectra at around room temperature and at 160 K for TBATFO, maleic acid, and their mixtures at various compositions.

The far-infrared spectra of selected mixtures containing maleic acid are depicted in Figure 10 at 160 and 320 K, together with the spectra of the pure components. Maleic acid presents an absorption peak around 170 cm^−1^, while TBATFO has its peak centered around 70 cm^−1^, due to the dispersion forces. At low temperatures, it becomes more structured, and a very evident structure around 80 cm^−1^ develops. In the mixtures, contrary to what has been observed in the mixtures with fumaric acid (Figure 7), one can see a broad band similar to that of TBATFO, without any evident frequency shift. This fact suggests a reduced variation of the dispersion forces in the mixtures with maleic acid. Interestingly, in the mixtures close to the eutectic one (30-70, 50-50, and 65-35), it is possible to observe the appearance of broad and poorly intense bands around 120 and 170 cm^−1^, which, due to their wavenumber, could be ascribed to the stretching mode of hydrogen bonds. This can be explained by the fact that the maleic acid in the mixtures develops HBs with TFO.

To sum up, the mixtures of TBATFO with fumaric acid display more evident marks of the interaction between the ionic couple and the acid than those mixtures with maleic acid: the OH stretching bands are displaced toward the higher frequencies, as well as showing C=O stretching. The νS=O asym is split, due to the interaction with the acid, and the asymmetric stretching of the CF_3_ group becomes broader. The vibrational modes of the carboxylic group, in particular δCOH, become shifted toward lower frequencies, due to the instauration of a different HB. In the far-infrared range, one observes a red shift of the peak, due to the dispersion forces between the alkyl chains. All these features are much less pronounced or are even absent in the mixtures with maleic acid.

This phenomenology can be explained by the fact that fumaric acid molecules have two possible sites for the formation of HB while, in maleic acid, one of the sites remains involved in intramolecular HBs, even after mixing. The interactions of fumaric acid with the ionic couple are stronger than those of maleic acid, leading to deeper changes in the mixtures with FUM. This picture is also consistent with the empirical observations of the appearance of the samples when heated to around 90 °C: while mixtures with FUM become liquid at high temperatures, the samples with MAL still retain a small fraction of solid component, as if the interactions among the components are not strong enough to create a homogeneous phase.

## 3. Materials and Methods

Tetrabutylammonium triflate (TBA-TFO), fumaric (FUM), and maleic acid (MAL), all with a purity of ≥99.0%, were purchased from Merck and were used without further purification. All components were solid at room temperature. The ionic solid and the acids were weighted in a proper molar ratio to obtain well-defined mixtures; typically, for each mixture, 2 g samples were prepared. The solid components were mixed by magnetic stirring at 80 °C for 1 h to obtain liquid phases. All samples were maintained in an inert argon atmosphere to avoid contamination from atmospheric air and water vapor. The compositions of the obtained mixtures will be indicated in the following section by their molar ratio. All mixtures were solid at room temperature.

To define the melting points of the pure components and of the mixtures, DSC measurements were conducted by means of a Star3 Mettler Toledo system, working down to −90 °C thanks to a Huber TC100 Crycooler. All measurements were performed on samples with a typical mass of 15-20 in a protective Ar flux of 40 mL/min, with a scanning rate of 5 °C/min.

Thermogravimetry measures were performed by means of a Setaram Setsys Evolution 1200 instrument with a temperature rate of 10 °C/min and a protective helium flux of 60 mL/min. A mass of about 10–20 mg was used for each sample.

Infrared absorbance spectra were collected in both the far- and mid-infrared range. The measurements in the wavenumber ranging between 30 and 680 cm^−1^ were collected at the AILES beamline of Synchrotron Soleil, by means of a IFS 125 HR spectrometer by Bruker (Billerica, MA, USA), a Si-coated mylar beamsplitter, and a bolometric detector. The temperature of the samples was varied by means of a Cryomec (Syracuse, NY, USA) pulse tube. The spectra in the wavenumber ranging between 450 and 6000 cm^−1^ were collected by means of an Agilent (Santa Clara, CA, USA) Cary 660 spectrometer, a DTGS detector, and a KBr beamsplitter. The temperature of the samples was modified by a Specac (Orpington, UK) Variable Temperature cell. For both types of measurements, the spectral resolution was fixed at 1 cm^−1^, and 100 scans were mediated for each spectrum. The spectra of the solid starting materials and of the mixtures containing fumaric acid were collected, with the powders dispersed in KBr or polyethylene in a mass ratio of 1:100 for the mid- and far-infrared range, respectively. The mixtures containing maleic acid were heated at 80 °C, where they displayed a partial liquid phase, and were then placed between two optical diamond windows of vacuum-tight cells. In both ranges, the samples were kept in a vacuum during measurement to avoid contamination from atmospheric gases and water vapor.

To allow for the interpretation of the infrared spectra and to evaluate the ionic coupling geometry, the structures of the TBA-TFO ion pairs and fumaric and maleic acids, as well as some combinations of the ion pairs and the acids, were computationally investigated. All calculations were performed using the Gaussian16 computational package [39]. The structure of each molecular species was investigated using density functional theory (DFT) calculations with the hybrid ωB97X-D functional [40], which includes long-range exchange correction (denoted by X), as well as semiclassical London-dispersion correction (indicated by suffix-D), and employing the standard 6-311G++(d,p) basis set. The good reliability of such a functional in describing the structural aspects and electronic properties of several liquids, considering non-covalent interactions, has been discussed in earlier reports [41]. The liquid environment was simulated by means of the polarized continuum model (PCM), using the parameters of 2-pentanone, which has a dielectric constant of 15 that is slightly higher than that typically found in ionic liquids [42,43]. IR spectra were obtained for each structure at the ωB97X-D/6-311++G** level, and vibrational assignment of the main absorption of each system was carried out on the basis of an analysis of the normal modes. To more deeply investigate the topological and energetic characteristics of the intermolecular interactions in each molecular complex, atoms in molecules (AIM) analysis was performed [44], employing the AIMAll software (Version 19.10.12) [45]. The nature of the interactions between components was investigated by analyzing the reduced-density gradient (RDG) [46] at low density, using the MultiWFN program [47]. Non-covalent interaction (NCI) analysis was carried out for the optimized clusters and the structures were visualized by the VMD program [48]. Fukui analysis [49] was carried out for each component to investigate the nucleophilicity and electrophilicity of the interacting groups.

## 4. Conclusions

The two isomers, fumaric and maleic acid, display different interactions with the ionic couple TBATFO. While mixtures with FUM give rise to an eutectic, mixtures with MAL retain a solid component, even at high temperatures. DFT computational investigation of the interaction suggests that TFO interacts more strongly with FUM, due to the availability of two sites for the formation of HB, while in MAL, one of the sites continues to be involved in intramolecular HBs. Infrared spectroscopy measurements support the stronger interaction of TBATFO with FUM; in the TBATFO-FUM mixtures, the SO_3_ and CF_3_ vibrational bands are split and displaced toward higher frequencies by the new HBs that form with the acid; in TBATFO-MAL mixtures, these bands are only slightly altered. In all cases, the OH bands of the acids are altered by HB interactions with the ionic couple. It is interesting to note that in the mixtures with FUM, the dispersion forces of the alkyl chains of the cation become weaker, while in the mixtures with MAL, one can observe weak bands due to the changes from the hydrogen bonds stretching.

## Figures and Tables

**Table 1:** Hydrogen bond geometry (Å) and topological analysis (a.u.), obtained at the ωB97XD/6-311++G** level for the different interaction models of FUM.

	FUM Figure 3a		FUM Figure 3b	
Hydrogen bond	OH⋅⋅⋅O=C		OH⋅⋅⋅O=C	
r (Å)	1.668		1.757	
ρ(r_c_) (a.u.)	0.0470		0.0354	
$\nabla^{2}$ ($r_{c}$) (a.u.)	0.1415		0.1273	
	**TFO + FUM** **Figure 3c**		**TFO + FUM** **Figure 3d**	
Hydrogen bond	OH⋅⋅⋅O=C	OH⋅⋅⋅O=S	OH⋅⋅⋅O=C	OH⋅⋅⋅O=S
r (Å)	1.657/1.679	1.661	1.722	1.608
ρ(r_c_) (a.u.)	0.0483/0.0456	0.0451	0.0391	0.0523
$\nabla^{2}$ ($r_{c}$) (a.u.)	0.1427/0.1385	0.1502	0.1337	0.1595
	**TBA + TFO + FUM** **Figure 3e**		**TBA + TFO + FUM** **Figure 3f**	
Hydrogen bond	OH⋅⋅⋅O=C	OH⋅⋅⋅O=S	OH⋅⋅⋅O=C	OH⋅⋅⋅O=S
r (Å)	1.661/1.667	1.663	1.713	1.601
ρ(r_c_) (a.u.)	0.0477/0.0470	0.0455	0.0401	0.0535
$\nabla^{2}$ ($r_{c}$) (a.u.)	0.1419/0.1406	0.1488	0.1361	0.1629

**Table 2:** Hydrogen bond geometry (Å) and topological analysis (a.u.) obtained at the ωB97XD/6-311++G** level for the different interaction models of MAL.

	MAL Figure 4c		
Hydrogen bond	OH⋅⋅⋅O=C intra	OH⋅⋅⋅O=C inter	OH⋅⋅⋅O=S
r (Å)	1.572 *^a^*	1.648/1.646	
ρ(r_c_) (a.u.)	0.0583 *^a^*	0.0441/0.0446	
$\nabla^{2}$ ($r_{c}$) (a.u.)	0.1600 *^a^*	0.1400/0.1426	
	**TFO + MAL** **Figure 4d**		
r (Å)	1.541/1.582	1.640	1.601
ρ(r_c_) (a.u.)	0.0676/0.0608	0.0495	0.0529
$\nabla^{2}$ ($r_{c}$) (a.u.)	0.1658/0.1622	0.14832	0.1604
	**TBA + TFO + MAL** **Figure 4e**		
r (Å)	1.555/1.593	1.651	1.664
ρ(r_c_) (a.u.)	0.0653/0.0588	0.0475	0.0469
$\nabla^{2}$ ($r_{c}$) (a.u.)	0.1642/0.1616	0.1472	0.144

*^a^* average value.

## Data Availability

Data are contained within the article or the Supplementary Materials.

## Supplementary Materials

The following supporting information can be downloaded at: https://www.mdpi.com/article/10.3390/molecules29215093/s1, Figure S1: Chemical structures of TBA, TFO, fumaric and maleic acids; Figure S2: TGA and DTA curves of maleic (panel a) and fumaric acids (panel b); Figure S3: C_2h_ symmetry structures of dimer (a), tetramer (b), hexamer (c), and octamer (d) of fumaric acid, calculated at the ωB97X-D/6-311++G(d,p) level; Figure S4: Complexes of TFO with fumaric acid (a) and TFO with a trimer of fumaric acid (b) calculated at B97X-D/6-311++G(d,p) level (Distance in Å); Figure S5: Nonamer of maleic acid calculated at B97X-D/6-311++G(d,p) level (Distance in Å); Figure S6: NIC of nonamer of maleic acid (isosurface=0.5); Figure S7: Scatter plot using promolecular density for MAL nonamer (a), FUM octamer (b) and TFO-MAL complexes of Figure 4d (c); Figure S8. Complexes of TFO with maleic acid (a) and TFO with a trimer of maleic acid (b) calculated at B97X-D/6-311++G(d,p) level (Distances in Å); Figure S9: DFT infrared spectra of the C_2h_ symmetry oligomers of FUM; Figure S10: DFT infrared spectra for TFO+FUM dimer and the TBA+TFO+FUM dimer complexes; Figure S11: DFT infrared spectra for TFO+MAL dimer and the TBA+TFO+MAL dimer complexes. Table S1: Second order perturbation theory analysis of Fock matrix of FUM complexes in NBO basis; Table S2: Second order perturbation theory analysis of Fock matrix of MAL complexes in NBO basis; Table S3: wb97xd/6-311++G** energy (Hartree) within the PCM method (Solvent = 2-pentanone).
