# Supplementary material for: Conformational Geometry Matters: The Case of the Low-Melting-Point Systems of Tetrabutylammonium Triflate with Fumaric or Maleic Acid
Source: Molecules. 2024 Oct 28;29(21):5093. doi: 10.3390/molecules29215093 (PMC11547753; doi:10.3390/molecules29215093)
Supplement: Supplementary file 1 [file molecules-29-05093-s001.zip › molecules-3256545-supplementary.pdf]

molecules

Supporting Information

Conformational Geometry Matters: The Case of the Low-Melting-Point Systems of Tetrabutylammonium Triflate with Fumaric or Maleic Acid

Simone Di Muzio [1,2], Fabio Ramondo [3], Oriele Palumbo [1,*], Francesco Trequattrini [1,4], Pascale Roy [5], Jean-Blaise Brubach [5] and Annalisa Paolone [1]

1 Istituto dei Sistemi Complessi, Consiglio Nazionale delle Ricerche, UOS La Sapienza, Piazzale Aldo Moro 5, 00185 Rome, Italy; simone.dimuzio@ifn.cnr.it (S.D.M.); francesco.trequattrini@uniroma1.it (F.T.); annalisa.paolone@roma1.infn.it (A.P.)
2 Istituto di Fotonica e Nanotecnologie, Consiglio Nazionale delle Ricerche, Piazza Leonardo da Vinci, 32, 20133 Milano, Italy
3 Department of Chemistry, Sapienza Università di Roma, Piazzale Aldo Moro 5, 00185 Rome, Italy; fabio.ramondo@uniroma1.it
4 Department of Physics, Sapienza Università di Roma, Piazzale Aldo Moro 5, 00185 Rome, Italy
5 Synchrotron SOLEIL, L'Orme des Merisiers, Départementale 128, 91190 Saint-Aubin, France; pascale.roy@synchrotron-soleil.fr (P.R.); jean-blaise.brubach@synchrotron-soleil.fr (J.-B.B.)
* Correspondence: oriele.palumbo@infn.roma1.it

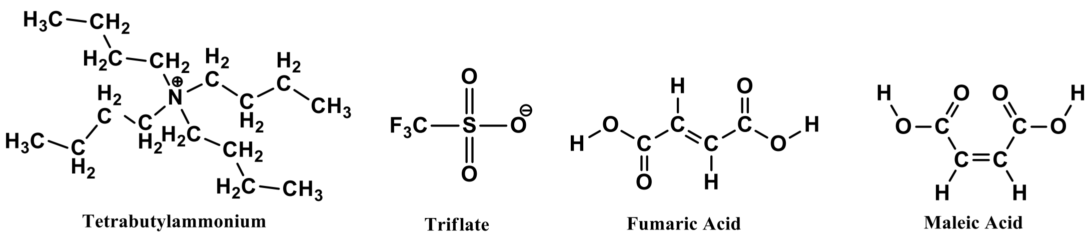

Figure S1. Chemical structure of TBA, TFO, Fumaric and Maleic acids.

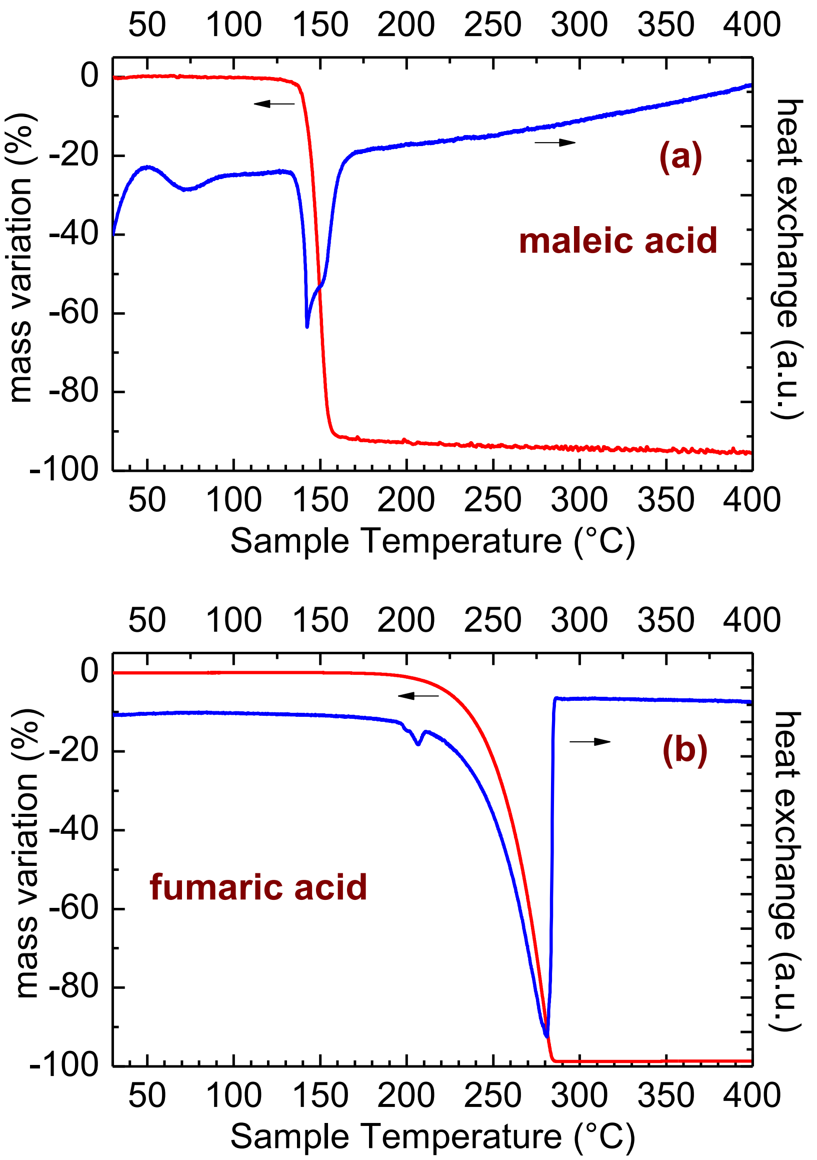

Figure S2. TGA and DTA curves of maleic (panel a) and fumaric acid (panel b).

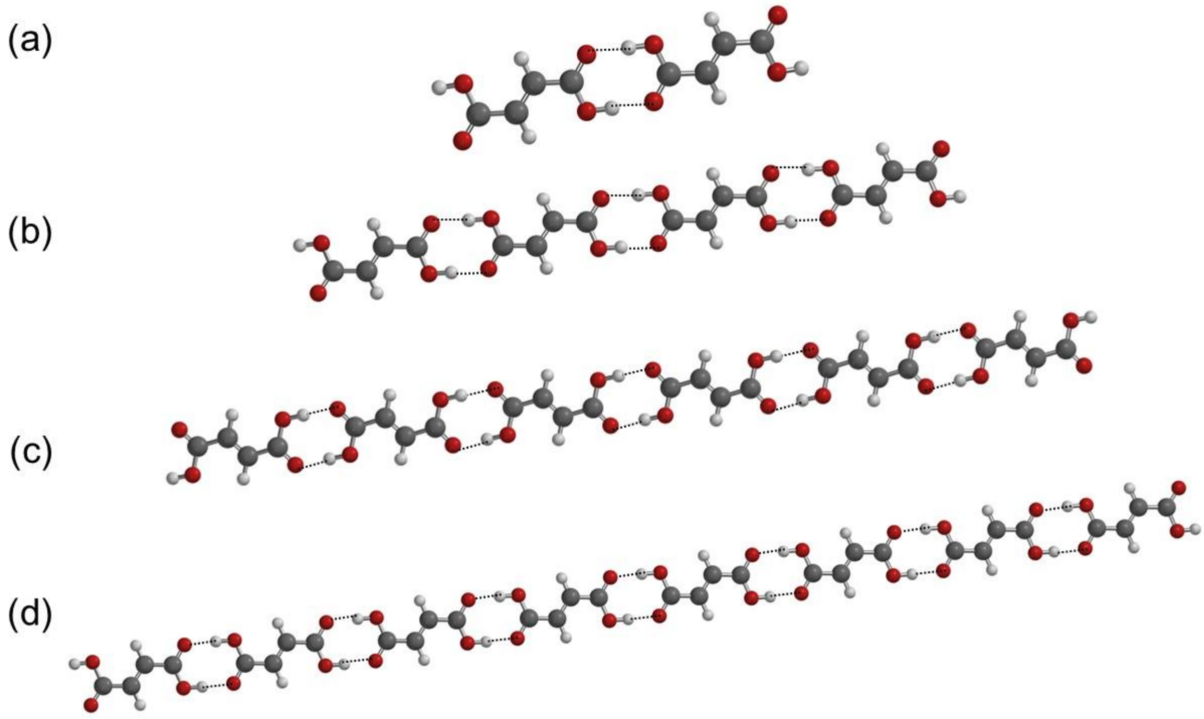

Figure S3. C_{2h} symmetry structures of dimer (a), tetramer (b), hexamer (c) and octamer (d) of fumaric acid calculated at ωB97X-D/6-311++G(d,p) level.

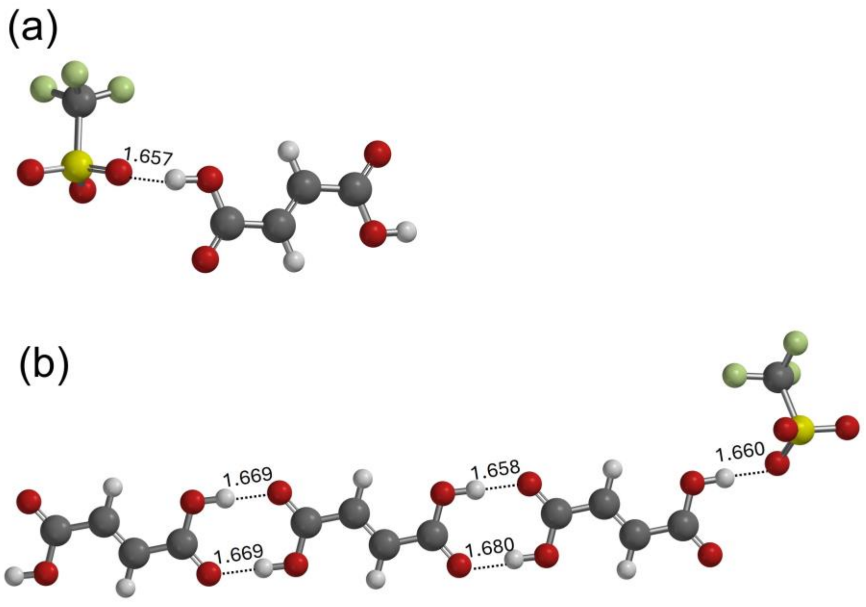

Figure S4. Complexes of TFO with fumaric acid (a) and TFO with a trimer of fumaric acid (b) calculated at ωB97X-D/6-311++G(d,p) level (Distance in Å).

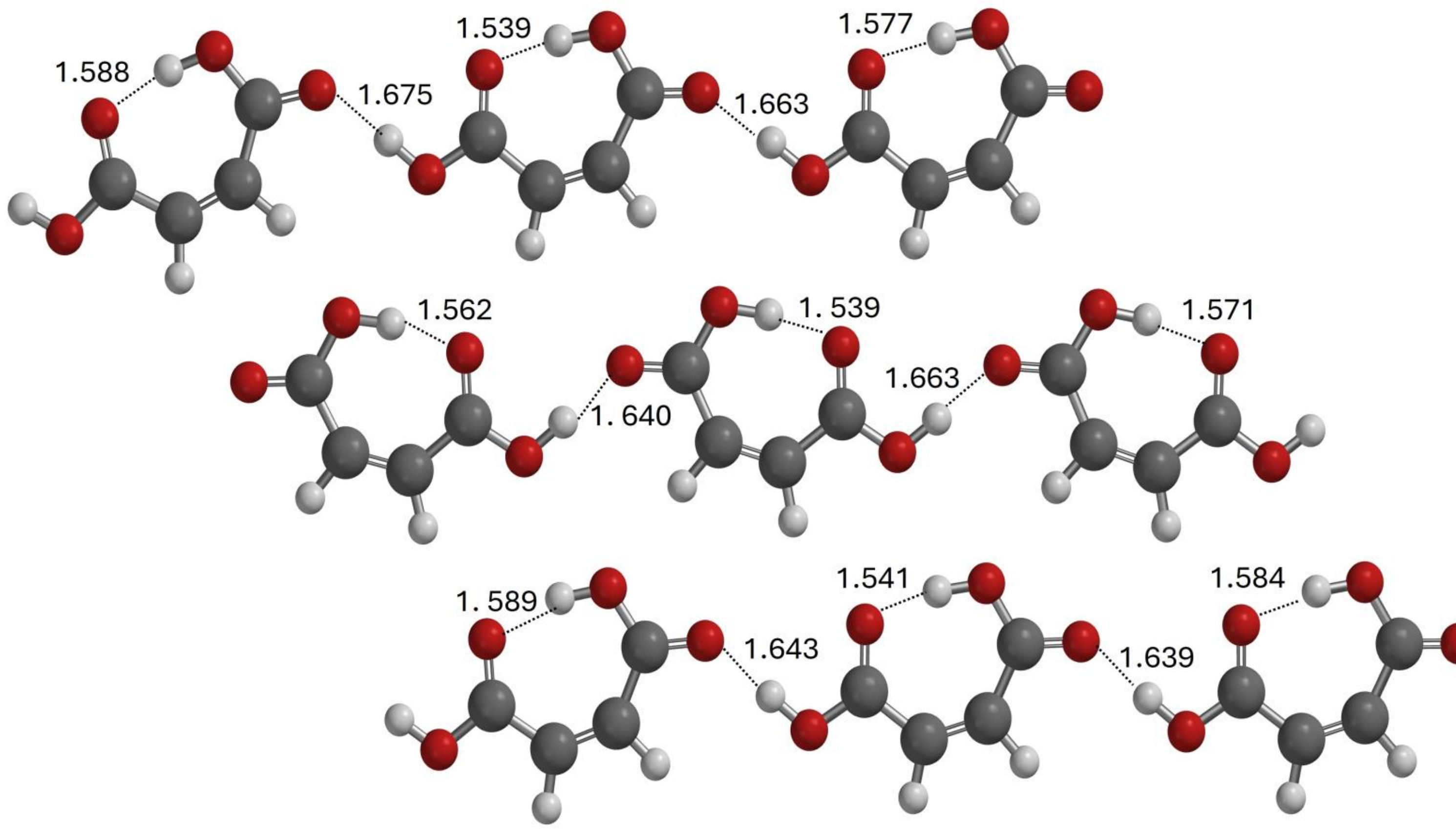

Figure S5. Nonamer of maleic acid calculated at ωB97X-D/6-311++G(d,p) level (Distance in Å)

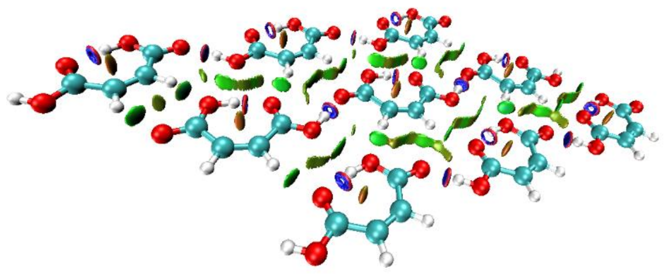

Figure S6. NIC of nonamer of maleic acid (isosurface=0.5).

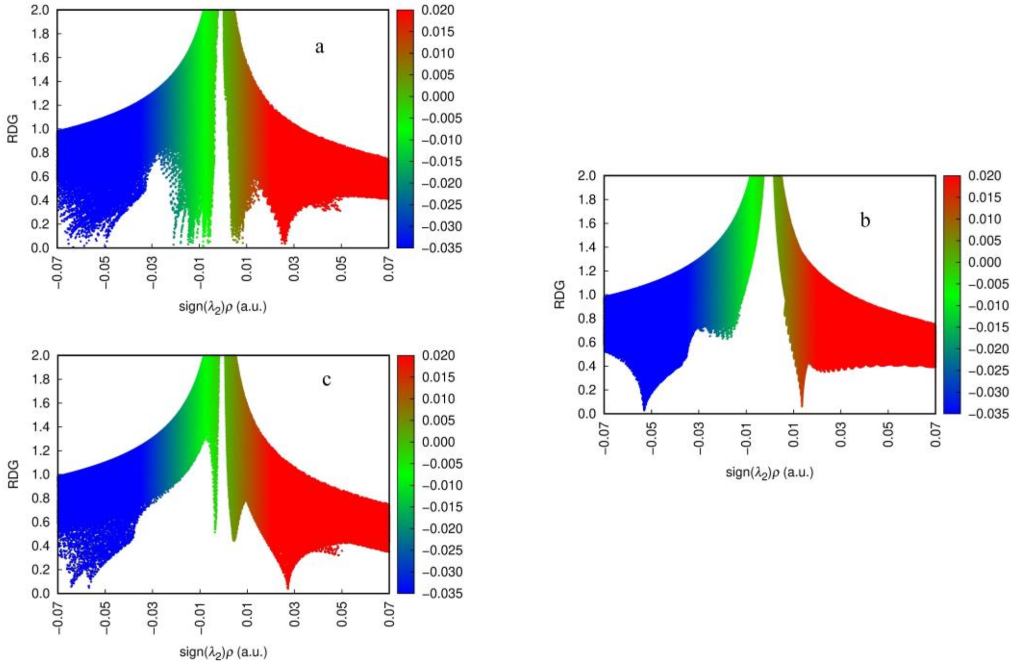

Figure S7. Scatter plot using promolecular density for MAL nonamer (a), FUM octamer (b) and TFO-MAL complexes of Fig. 4d (c).

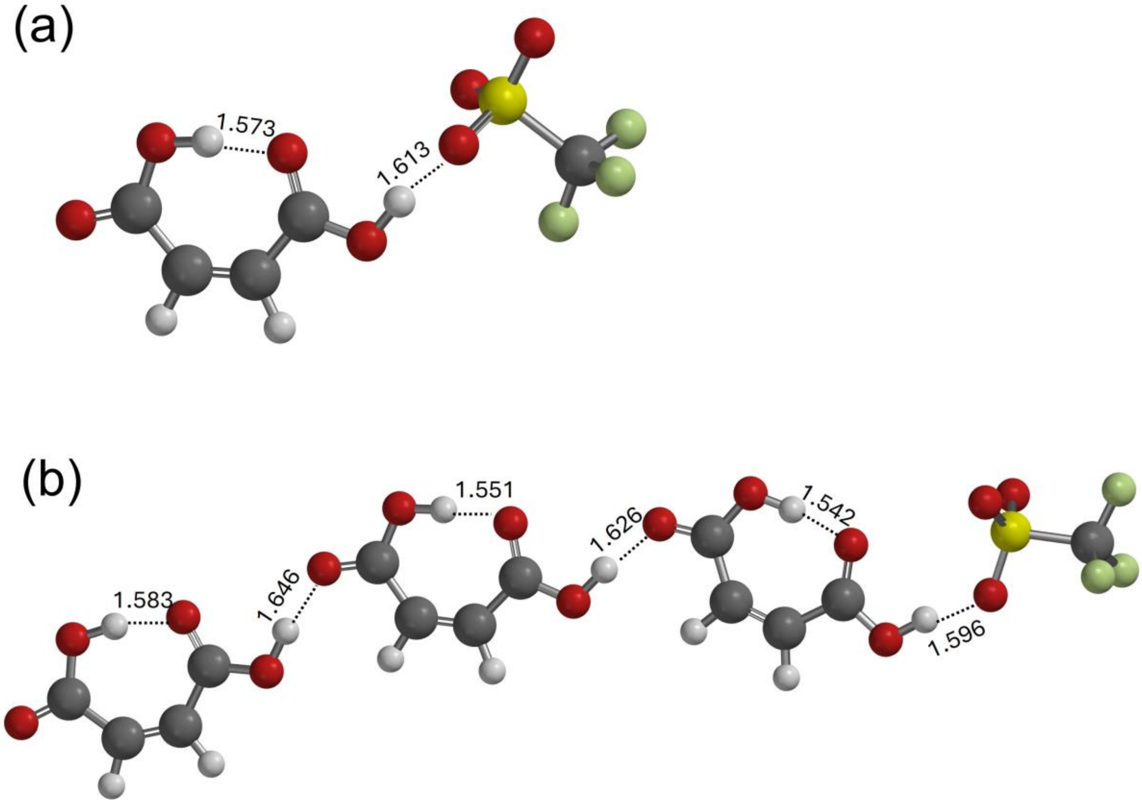

Figure S8. Complexes of TFO with maleic acid (a) and TFO with a trimer of maleic acid (b) calculated at ωB97X-D/6-311++G(d,p) level (Distances in Å).

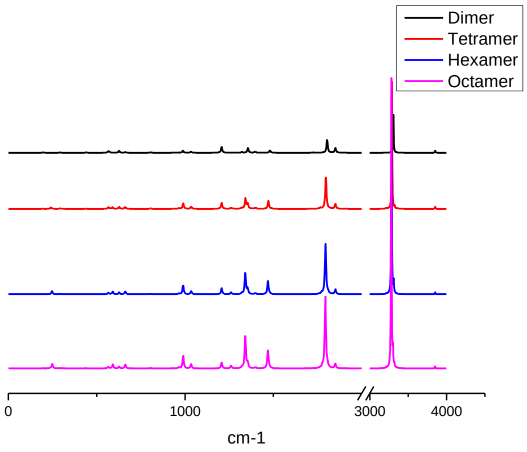

Figure S9. DFT infrared spectra of the C_{2h} symmetry oligomers of FUM.

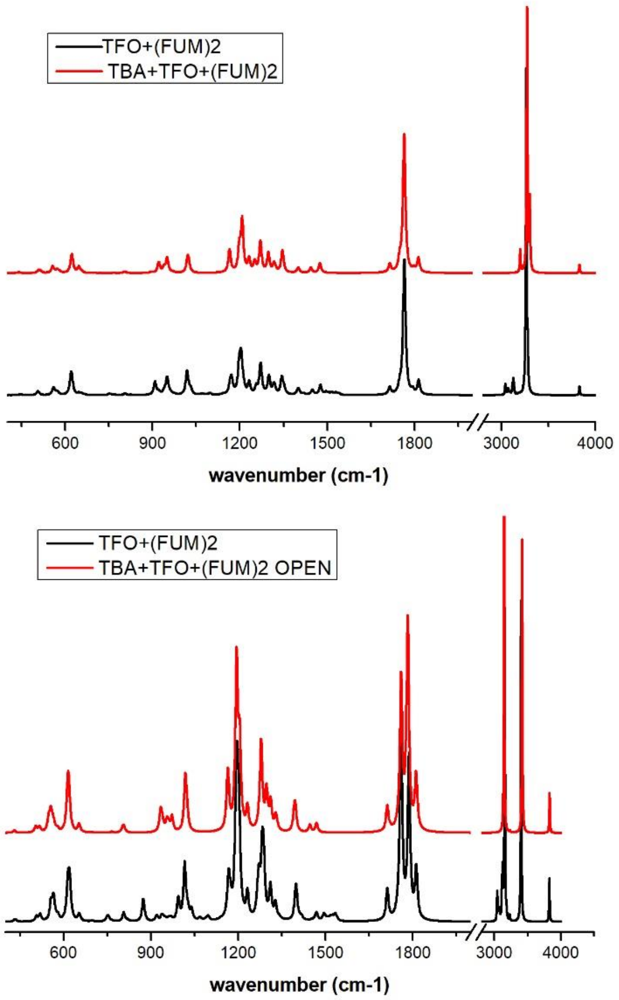

Figure S10. DFT infrared spectra for TFO+FUM dimer and the TBA+TFO+FUM dimer complexes.

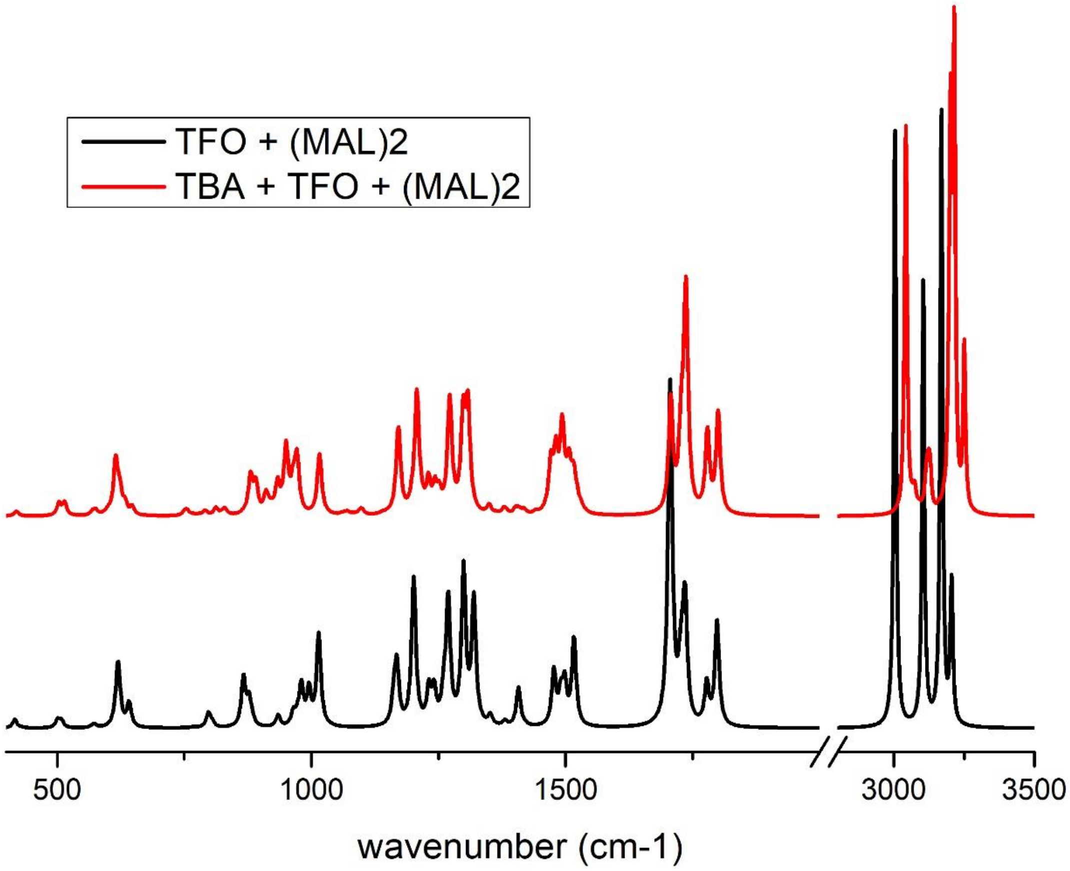

Figure S11. DFT infrared spectra for TFO+MAL dimer and the TBA+TFO+MAL dimer complexes.

Table S1. Second order perturbation theory analysis of Fock matrix of FUM complexes in NBO basis..

Donor (i)	Acceptor (j)	$E^{(2)\,a}$	E(i) – E(j)[b]	F(i,j)[c]
FUM (Fig. 3a)				
LP(O)	σ*O-H	24,60	0,87	0,133
LP(O)	σ*O-H	24,60	0,87	0,133
FUM (Fig. 3b)				
LP(O)	σ*O-H	10,55	0,89	0,089
TFO+FUM (Fig. 3c)				
LP(O)	σ*O-H	28,31	0,87	0,142
LP(O)	σ*O-H	22,65	0,88	0,129
LP(O)TFO	σ*O-H	12,62	0,86	0,095
TFO+FUM (Fig. 3d)				
LP(O)	σ*O-H	12,59	0,89	0,097
LP(O)TFO	σ*O-H	7,67	0,84	0,075

[a] $E^{(2)}$ indicates the energy of hyperconjugate interactions (stabilization energy) (kcal/mol)

[b] Energy difference between donor and acceptor i and j NBO orbitals

[c] F(i,j) is the Fock matrix element between i and j NBO orbitals

Table S2. Second order perturbation theory analysis of Fock matrix of MAL complexes in NBO basis..

Donor (i)	Acceptor (j)	$E^{(2)\,a}$	E(i) – E(j)[b]	F(i,j)[c]
MAL dimer				
LP(O)	σ*O-H[d]	34,47	0,90	0,161
LP(O)	σ*O-H[d]	34,45	0,90	0,161
LP(O)	σ*O-H[e]	23,61	0,87	0,131
TFO+MAL (Fig. 4d)				
LP(O)	σ*O-H[d]	44,94	0,88	0,181
LP(O)	σ*O-H[d]	36,08	0,90	0,164
LP(O)	σ*O-H[e]	28,04	0,86	0,142
LP(O)TFO	σ*O-H	17,63	1,32	0,137

[a] $E^{(2)}$ indicates the energy of hyperconjugate interactions (stabilization energy) (kcal/mol)

[b] Energy difference between donor and acceptor i and j NBO orbitals

[c] F(i,j) is the Fock matrix element between i and j NBO orbitals

[d] OH connected by intramolecular hydrogen bond.

[e] OH connected by intermolecular hydrogen bond.

Table S3. ωb97xd/6-311++G** energy (Hartree) within the PCM method (Solvent=2-pentanone)

TFO-TBA	-1647.631026

FUMARIC ACID

Monomer	-455.7435578
Dimer (Fig. 3a)	-911.5061604
Dimer (Fig. 3b)	-911.4943210
Tetramer (Fig. S3b)	-1823.010013
Hexamer (Fig. S3c)	-2734.529433
Octamer (Fig. S3d)	-3646.048922

TFO+FUMARIC ACID

FUM Monomer (Fig. S4a)	-1417.381723
FUM dimer (Fig. 3c)	-1873.145459
FUM dimer (Fig. 3d)	-1873.139423
FUM trimer (Fig. S4b)	-2328.909086

TBA+TFO+FUMARIC ACID

FUM dimer (Fig. 3e)	-2559.158832
FUM dimer (Fig. 3f)	-2559.157773

MALEIC ACID

Monomer	-455.7407504
Dimer	-911.4939478
Trimer (Fig. 4a)	-1367.247728
Hexamer (Fig. 4b)	-2734.512966
Nonamer (Fig. 4c)	-4101.776939

TFO+MALEIC ACID

MAL monomer (Fig. S5a)	-1417.382338
MAL dimer (Fig. 4d)	-1873.136283
MAL trimer (Fig. S5b)	-2328.890207

TBA+TFO+MALEIC ACID

MAL dimer (Fig. 4e)	-2559.154911

Disclaimer/Publisher's Note: The statements, opinions and data contained in all publications are solely those of the individual author(s) and contributor(s) and not of MDPI and/or the editor(s). MDPI and/or the editor(s) disclaim responsibility for any injury to people or property resulting from any ideas, methods, instructions or products referred to in the content.
